# Correlation of Bony Invasion With Nodal Metastasis, Pattern of Invasion and Survival in Oral Squamous Cell Carcinoma: A Retrospective Analysis of 122 Primary Cases From Oral Cancer Centre of South India

**DOI:** 10.7759/cureus.42887

**Published:** 2023-08-03

**Authors:** Swetha Ramasubramanian, Deepak Pandiar, Reshma P Krishnan, Karthikeyan Ramalingam, Ronell Bologna-Molina

**Affiliations:** 1 Dentistry, Saveetha Dental College and Hospitals, Saveetha Institute of Medical and Technical Sciences, Saveetha University, Chennai, IND; 2 Oral Pathology and Microbiology, Saveetha Dental College and Hospitals, Saveetha Institute of Medical and Technical Sciences, Saveetha University, Chennai, IND; 3 Diagnostics in Oral Pathology and Oral Medicine, Facultad de Odontología, Universidad de la República, Montevideo, URY

**Keywords:** survival, squamous cell carcinoma, oral, nodal metastases, bone invasion

## Abstract

Background

Oral squamous cell carcinoma (OSCC) is considered to be the most common epithelial malignant neoplasm of the oral cavity. Despite advancements in diagnosis and therapeutics the clinical outcome of the disease has not improved much which may be attributed to tumor biology and heterogeneity. Bone invasion by cancer cells is currently staged as a moderately advanced disease. However, many low-grade carcinomas such as verrucous carcinoma and carcinoma cuniculatum show body invasion but less nodal metastases and better overall survival. The present study was orchestrated to analyze if bone invasion in OSCC has any impact on regional nodal metastases and survival.

Materials and methods

A total of 122 cases of OSCC who underwent excision and neck dissection were retrieved and included. These cases were then divided into two study groups. Group I comprised 56 OSCC cases with bone involvement and 66 cases with no bony involvement. The bone invasion was correlated with nodal metastases, survival and pattern of invasion. Statistical analysis was done using SPSS software (IBM Corp., Armonk, NY, USA).

Results

There was no statistically significant correlation between bone invasion with either nodal metastases or pattern of invasion, however, the worst pattern of invasion (WPOI)-4,5 showed a statistically higher incidence of nodal involvement in OSCC. No statistical difference was noted in overall survival between the two groups.

Conclusion

The worst pattern of invasion and not bone involvement, depicts nodal metastases in OSCC and thus, deserves consideration while staging and treatment planning.

## Introduction

Head and neck cancer is a group of heterogeneous malignancies mainly comprised of oral, oropharyngeal, pharyngeal, and laryngeal squamous cell carcinoma, and among these, 90% of the cases are constituted by oral squamous cell carcinoma (OSCC) [1,2]. The survival rate of patients with lymph node metastasis (LNM) following surgery has previously been reported as 20-30% as compared to 63-86% in LNM-free patients implying that regional nodal metastasis is an important prognosticator in OSCC [3]. The primary characteristic of malignant cells is their ability to dissociate and invade adjacent structures and distant sites attributing to their spread to the distant sites through lymphatic and hematogenous routes [4,5]. Among many prognostic factors, the size of the primary tumor bears significant prognostic value. As the tumor increases in size, not only does it invade the surrounding tissue but also shows a propensity to metastasize to regional neck nodes, however, distant metastases are rarely encountered in OSCC [6]. Many clinical and histopathologic parameters have been proposed for the survival of OSCC patients such as site, extension, tumor stage and grade, tumor thickness, and perineural invasion [7].

In addition to grading, some studies have also attempted to determine if any other histologic parameter correlates with aggressive biological behavior. Spiro et al. [8] demonstrated that OSCC patients with a worse pattern of invasion had more propensity to present regional and distant metastases and mortality. According to the latest TNM classification (AJCC Cancer Staging Manual, 8th edition) involvement of the underlying bone (maxilla/mandible) upstages the tumor grading however, this finding cannot be generalized owing to the fact that there is a marked difference in the sites affected between the eastern and western countries [9-11]. This is further contributed to differences in deleterious habits. In the previous AJCC grading system (7th edition), involvement of extrinsic muscle of the tongue was also staged as T4a, however, it was excluded in the 8th edition [12]. In particular, the tongue is not the most common site of involvement in South Asian countries, where the commonest site is the gingivobuccal complex.

Thus, bony involvement could possibly be an inevitable event in South Asian countries as compared to tongue carcinoma, which is more frequently seen in the Eastern part of the world. Many studies have emphasized on depth and pattern of invasion, perineural invasion but there are limited studies correlating bony invasion with the metastatic behavior of oral squamous cell carcinoma with conflicting results, thus the present study aimed to correlate bony invasion and pattern of invasion with loco-regional nodal metastases.

## Materials and methods

The present retrospective study was conducted in the Department of Oral Pathology and Microbiology and included a total of 122 consecutive primary oral squamous cell carcinoma cases which were treated with surgery and neck dissection with or without adjuvant therapy. All the included cases were initially histologically diagnosed as OSCC on incisional biopsy. Further, the cases where the haematoxylin and eosin (H&E)-stained slides or blocks (formalin fixed paraffin embedded) were not available for histological examination were excluded, as in cases where the patients chose to opt for other cancer centres for further treatment. The cases were reviewed by SR (first author) and the slides of the excised specimen including the lymph nodes were re-analyzed by DP (second author). Prior approval was obtained from the institutional ethical clearance board (IHEC/SDC/OPATH-1924/22/012).

The descriptive data of these patients were retrieved from the electronic database of the institution and included age, gender, laterality, location, histological grade, and stage. The slides were re-examined for the involvement of bone, nodal status, and worst pattern of invasion (WPOI) at the invasive tumor front (ITF). The cases were finally divided into two groups. Group I consisted of 56 cases with histological evidence of bone involvement (pathological T4a). Superficial cortical erosion was not considered as bone involvement. The remaining 66 were classified as Group II, where the bony involvement was not seen histopathologically. Frank invasion by tumor cells beyond the cortices of maxilla and mandible into the medullary spaces, grossly and histologically, was reported as bone invasion.

**Figure 1:**
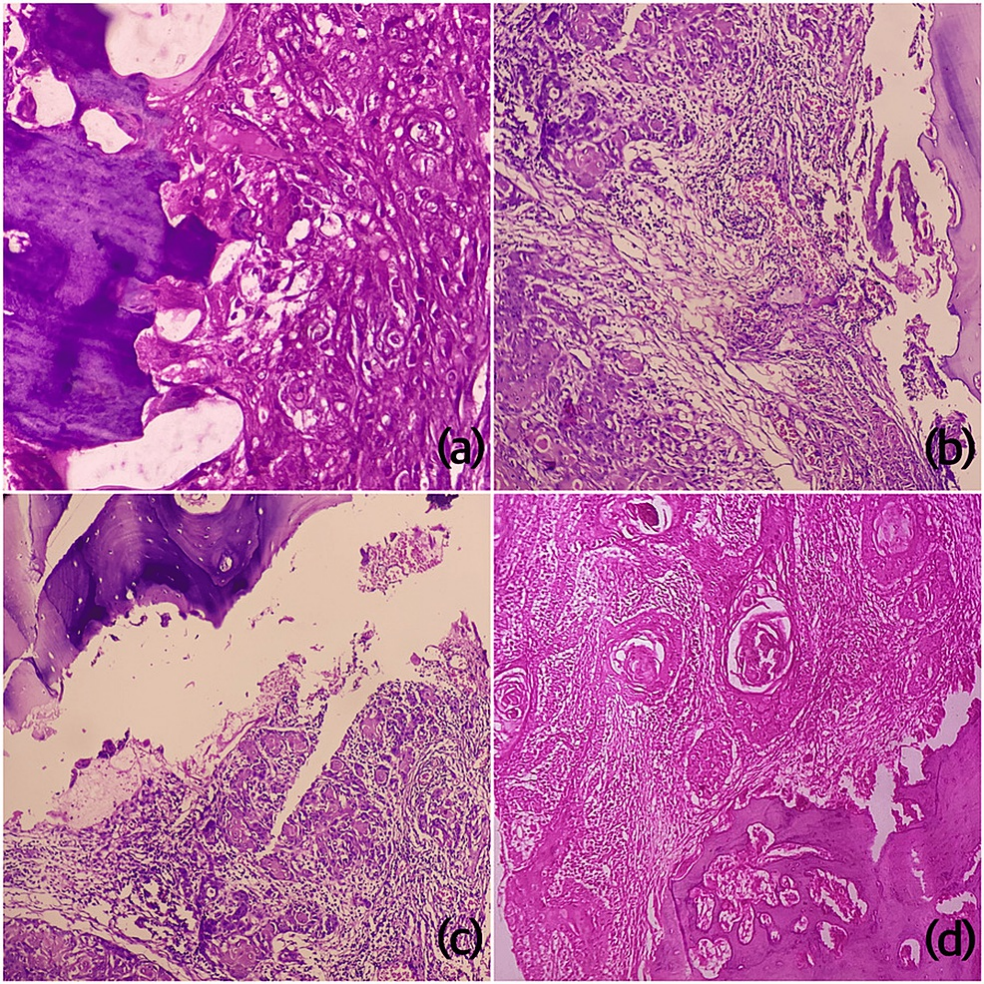
Photomicrographs of H&E-stained sections showing different patterns of bony invasion by OSCC (a-c, WPOI 4; d, WPOI 1) H&E: haematoxylin and eosin; WPOI: worst pattern of invasion; OSCC: oral squamous cell carcinoma.

The histological grading was done using Bryne’s grading system [13]. Tumor grading was done at the invasive tumor front (ITF) which considers four parameters, namely, the degree of keratinization, pattern of invasion (POI), intensity of inflammatory infiltrate and nuclear pleomorphism. In this system, a score of 1 to 4 is given to each parameter; 1 is the lowest score and 4 is the highest score. Finally, the scores are summed up and based on the malignancy scores, the cases were divided into three prognostic groups as follows: a) well-differentiated squamous cell carcinoma (score: 4-8), b) moderately differentiated squamous cell carcinoma (score: 9-12), and c) poorly differentiated squamous cell carcinoma (score: 13-16). Almangush et al. [14] modified this scoring system by adding 5th type of pattern of invasion. POI was assessed for all the cases at ITF. POI 1-3 were considered as better POI and were clubbed together while POI 4 and 5 were considered as worst patterns of invasion (Figure 1).

​​The results were entered in a Microsoft Excel spreadsheet (2021). Data were analyzed using IBM SPSS software (IBM Corp., Armonk, NY, USA). For frequency counts, descriptive statistics were done. Comparison between the two groups was made using Chi‐square test. A p-value of < 0.05 was considered to be statistically significant. Kaplan-Meier graphs were used to plot survival curves and the Log rank test was used to compare Group I and Group II. The event of interest was death due to any cause.

## Results

Clinico-demographic profile

In total, 122 histologically proven cases of OSCC were included in the present study. All cases underwent excision with neck dissection. Overall, males outnumbered females with a ratio of 3.7:1 (96M:26F). The cases were divided into two study groups. Group I comprised 56 cases of OSCC with gross and histological infiltration into the medullary bone (c/pT4a) of which 47 were males and nine were females (M: F::5:1). The mean age of occurrence was 50 years (±9.6; Range 35-68 years). In group II (no bony involvement), there were 66 cases (49 males, and 17 females; M: F::2.8:1). The mean age of occurrence was 54.326 years (±11.151; Range 27-75 years). No significant difference was found in mean age, gender, and laterality between the two study groups (p > 0.05). A detailed description is given in Table 1.

**Table 1:** Clinicopathological profile of the patients in group I and group II # statistically not significant, * statistically significant

Parameter	Group I	Group II	p-value
Number of cases (Total 122)	56	66	
Age	Mean: 50±9.6 years, Median: 49 years, Range: 35-68 years	Mean: 54.326±11.151 years, Median: 53.5 years, Range: 27-75 years	0.64^#^
Gender	Male: 47, Female: 9, M:F: 5:1	Male: 49, Female: 17, M:F: 2.8:1	0.08^#^
Laterality	Left: 26, Right: 30	Left: 29, Right: 37	0.31^#^
Site	Gingiva and gingivobuccal sulcus: 33, Buccal mucosa: 17, Retromolar trigone: 3, Lateral border of tongue: 3	Gingiva and gingivobuccal sulcus: 14, Buccal mucosa: 37, Retromolar trigone: 3, Lateral border of tongue: 12	0.04^*^

Gingiva and gingivobuccal sulcus (GBS) were significantly more commonly affected sites followed by buccal mucosa in Group I and a reverse trend was noted in Group II. Out of all the included cases, only one case showed distant metastases, initially to the lungs and then widespread visceral metastasis. This patient was staged as T4a, the tumor involved the facial skin, however, no bony involvement was noted. This patient succumbed to the disease within a few months post-treatment.

Correlation of bone invasion with nodal metastasis

In group I, 50% of the cases showed regional nodal metastasis and the remaining 50% (28 cases each) were pathologically N0. In contrast, 56.1% of patients (37/66) in group II showed no regional metastases and the remaining 43.9% cases (29/66) with no histopathological evidence of bone invasion had nodal metastases. No statistically significant difference was noted between bone invasion by tumor cells and nodal metastases (p-value: >0.05) (Table 2).

**Table 2:** Correlation of bone invasion by OSCC with regional nodal metastases and worst pattern of invasion (WPOI) # statistically not significant OSCC: Oral squamous cell carcinoma

	No regional nodal metastasis (N_0_)	With regional nodal metastasis		WPOI 1-3	WPOI 4-5	
Group I (OSCC with bone invasion; n=56)	28 (50%)	28 (50%)	p > 0.05^#^	34 (60.7%)	22 (39.3%)	p > 0.05^#^
Group II (OSCC without bone invasion; n=66)	37 (56.1%)	29 (43.9%)		36 (54.54%)	30 (45.46%)	

Correlation of bone invasion with the worst pattern of tumor invasion

The bony invasion was also correlated with the worst pattern of tumor invasion. In cases with bone invasion (Group I), 22 cases (39.3%, 22/56) showed WPOI- 4 and 5, while the rest 60.7% (34/56) cases showed a better pattern of invasion (WPOI 1-3). Notably, POI 1-3 demonstrated more incidence of bony involvement than POI 4-5. In group II (without bone invasion), 45.46% of cases (30/66) showed WPOI-4,5 and 54.54% (36/66) showed WPOI-1,2,3. There was no statistically significant difference between the pattern of tumor invasion and medullary involvement of bone (p-value: >0.05) (Table 2).

Correlation of worst pattern of tumor invasion with nodal metastasis

Further, the pattern of invasion was correlated with loco-regional metastasis. Sixty-seven cases had WPOI 1/2/3 and 55 cases showed WPOI-4/5. The cases with WPOI 1/2/3 showed nodal metastases in 29.85% of cases (20/67) and no regional metastases in the remaining 70.15% of cases (47/67). Contrary to this, 46/55 cases (83.6%) with WPOI 4/5 showed regional metastases to the cervical lymph nodes and the remaining 9/55 (16.4%) were pathologically N0. There was a statistically significant difference between the worst pattern of invasion and nodal metastases (p-value: <0.001) (Table 3).

**Table 3:** Correlation of regional nodal metastases in OSCC with the worst pattern of invasion (WPOI). * statistically significant OSCC: Oral squamous cell carcinoma

	With regional nodal metastasis	No regional nodal metastasis (N_0_)	
WPOI 1-3 (n=67)	20 (29.85%)	47 (70.15%)	P<0.001^*^
WPOI 4-5 (n=55)	46 (83.6%)	6 (16.4%)	

Treatment and survival

**Figure 2:**
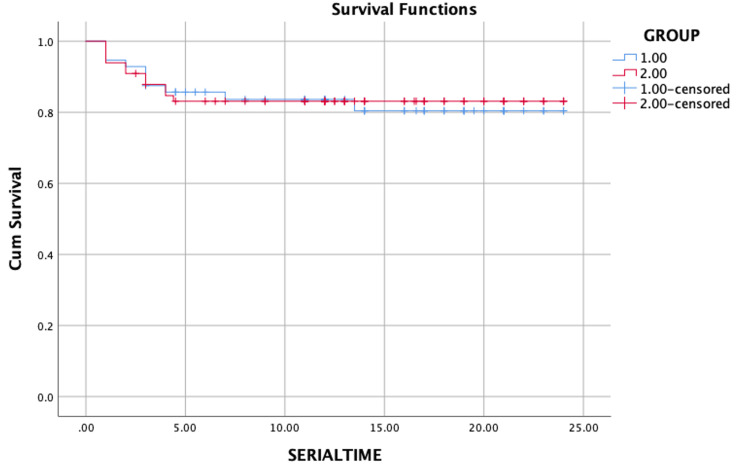
Kaplan–Meier curve for overall survival stratified by bone involvement in oral squamous cell carcinoma

After discussion in the institutional tumor board, all the patients underwent wide excision with radical neck dissection with or without chemo- and radiotherapy. The maximum follow-up period was 24 months. Overall survival was 20.282 months. The mean survival in group I and group II were 20.201 months and 20.358 months, respectively. The bone invasion did not affect overall survival in patients with OSCC (p-value: 0.903) (Figure 2).

## Discussion

The burden of oral cancer particularly in developing nations like India is staggeringly high and cases from India alone comprise about one-third of the OSCC patients worldwide [15]. Moreover, OSCC in India accounts for 30% of all cancers [15]. There is drastic variation in the anatomical sites involved by OSCC in the Eastern and Western world. While, in the West, the tongue is the most commonly affected site, buccal mucosa, and GBS are frequently involved in the Indian subcontinent which is further dependent on the variation in the habits. According to the latest AJCC staging system, bone invasion by OSCC is designated as a moderately advanced disease and staged as c/pT4a. However, unlike the previous edition, the involvement of extrinsic muscles of the tongue is no longer considered a staging criterion for T4 designation, owing to the fact that depth of invasion (DOI) supersedes extrinsic muscle infiltration. Akin to this, bone invasion in the OSCC of other oral sites particularly GBS and alveolus can occur even in tumors with smaller size. Nodal metastasis is an important predictor of a patient’s disease-free survival and overall survival and ultimately the treatment. Thus, the present study was orchestrated to assess if the involvement of bone in OSCC correlates with locoregional metastasis and survival.

In the present study, we did not find any significant correlation between medullary invasion by tumor cells and incidence of cervical lymph nodes, neither did the bony invasion correlate with the type of invasion pattern and survival. The impact of bone invasion has been studied previously with contrasting results [16,17]. Unlike tongue SCC, where the bone invasion occurs later in the disease progression, OSCC of other anatomical sites such as gingiva, tends to infiltrate the underlying bone even when the size of the tumor is small. Furthermore, other variants such as verrucous carcinoma and carcinoma cuniculatum, both of which are considered as low-grade tumors, show a propensity for bony invasion in the absence of nodal metastasis [18,19]. The enlarged lymph nodes are often reactive. This raises a very important query about whether such tumors qualify to be designated as moderately advanced.

Bone involvement in OSCC has a complex molecular mechanism. Three types of invasive patterns have been explained for OSCC which involves the bone viz., erosive, infiltrative, and mixed which have been correlated with clinical behaviors. The erosive pattern is not considered moderately advanced in the current staging system. It has been demonstrated that the tumors which show infiltrative bone invasion show higher expression of tumor necrosis factor-alpha (TNF-µ), interleukins (IL-6 and 11), and parathyroid hormone-related protein (PTHrP) as compared to tumors with superficial erosion of the cortical bone [20]. These cytokines further cause either suppression of osteoprotegerin (OPG) or expression of RANKL in both the stromal cells and OSCC cells inducing osteoclastogenesis [21]. Cumulatively, a microenvironment is generated by oral squamous cell carcinoma for osteoclastogenesis and subsequent resorption implying a difference in the tumor biology.

In previous studies, the worst pattern of invasion has been correlated with disease outcome and a poor prognosticator in various human malignancies such as colorectal, oral, pancreatic, oesophageal, gall bladder, and ampulla [22-25]. Oral squamous cell carcinoma is histologically graded into well, moderately, and poorly differentiated, and it has been repeatedly demonstrated that Bryne’s grading system, which includes the pattern of invasion as an important parameter for grading, bears a prognostic value than the WHO grading system [26]. We divided WPOI broadly into two categories (WPOI 1-3 - cohesive and WPOI 4-5 - non-cohesive). Mishra et al. [27] found WPOI as an independent prognosticator in oral squamous cell carcinoma and significantly correlated with advanced tumor stage, poor tumor differentiation, perineural invasion, poor survival, more DOI, and higher locoregional metastasis. In the present study, we correlated WPOI with bone invasion and nodal metastasis and found that WPOI did not correlate with the involvement of bone, however, a statistically significant difference was seen between the WPOI and nodal metastasis, which is an independent prognosticator for overall survival in oral squamous cell carcinoma. While in gingival or GBS SCC, the phenomenon appears to be locally infiltrative, it is actually moderately advanced when the other oral soft tissues are involved such as tongue or buccal mucosa alone, which in comparison are anatomically distant from the mandible and maxilla. In the present study, we found that the cases with WPOI 4-5 showed more propensity for nodal metastasis in contrast to cases with better patterns of invasion, which displayed a tendency for local infiltration. More than bony invasion, other factors dictate recurrence and metastasis in OSCC such as DOI, size of tumor cells at ITF, calibre of lymphatic channels, angiogenesis, perineural invasion, and surgical margins to name a few. Thus, the involvement of bone alone does not appear to suffice to be graded as T4a particularly in a few anatomical sites and in the Eastern population, where the pattern of invasion deserves consideration rather.

Although the present study included a smaller sample size, it does draw a few important points. Firstly, whether the bone involvement in oral squamous cell carcinoma of any location be considered as c/pT4a? Secondly, apart from tumor size and DOI, should the pattern of invasion be considered a parameter in the current staging system? More elaborative studies with larger sample size from South Asian nations are warranted to validate the results.

## Conclusions

Oral squamous cell carcinoma is a lethal disease with grave outcomes. The present study was conducted to assess the role of bone invasion and pattern of invasion on nodal metastasis and survival in OSCC patients. It was found that WPOI and not bony involvement are correlated with lymph node metastasis. Further, bone invasion does not appear to affect the overall survival in patients with OSCC. More elaborative and multicentric studies with more samples are required for validation.
